# Supplementary material for: Determining the Optimal Vacuum Frying Conditions for Silver Herring (Spratelloides gracilis) Using the Response Surface Methodology
Source: Foods. 2023 Sep 22;12(19):3533. doi: 10.3390/foods12193533 (PMC10572491; doi:10.3390/foods12193533)
Supplement: Supplementary file 1 [file foods-12-03533-s001.zip › foods-2629931-supplementary.pdf]

Table S1. Regression coefficients and analysis of variance (ANOVA) of the response as a function of the independent variables on vacuum fried silver herring.

Y	Aw	mositure content (%)	yield (%)	oil content (%)	L^* value	ΔE	fracturability (g/s)
X_1	-0.12	-0.9	-2.09	-1.1	1.73	1.72	-16.13
X_2	-0.051	-0.34	7.50E-03	1.47	-0.3	-0.3	27.27
X_3	0.024	0.35	3.53	-0.88	-0.5	-0.53	76.29
$X_1 X_2$	0.057	-0.42	0.64	1.06	-0.91	-0.92	5.79
$X_1 X_3$	-3.25E-03	0.12	-0.33	-1.15	-0.42	-0.41	74.32
$X_2 X_3$	-6.83E-03	-0.28	0.79	-0.19	0.36	0.36	9.37
X_1^2	0.092	0.44	0.35	1.17	-0.062	-0.094	36.2
X_2^2	0.077	0.25	0.79	1.59	0.39	0.4	29.27
X_3^2	-0.04	0.14	0.33	-1.4	-1.11	-1.1	41.85
Model (F-Value)	5.16	4.16	4.45	5.95	4.53	4.44	2.86
Model (P-value)	0.0428	0.0917	0.0575	0.0319	0.0554	0.0576	0.13
R^2	0.9028	0.9034	0.889	0.9146	0.8908	0.8888	0.8373
Lack of Fit (P-value)	0.0526	0.1554	0.0585	0.744	0.8873	0.9056	0.524

Table S2. The experimental results of the central composite rotatable design for VF silver herring.

Run	VF temperature (°C)	VF duration (min)	maltose concentration (%)	Aw	moisture content (%)	yield (%)	oil content(%)	L^* value	ΔE	fracturability (g/s)
1	75	25	15	0.63±0.01	7.7±0.23	25.24±2.11	27.78±2.47	24.65±1.43	24.82±1.23	279±18.34
2	105	25	15	0.21±0.01	2.8±0.40	20.06±1.95	23.66±1.49	30.56±1.80	30.73±1.71	342±25.12
3	75	45	15	0.34±0.01	4.47±0.29	26.84±1.45	29.62±1.77	25.89±1.49	26.14±1.57	289±39.78
4	105	45	15	0.14±0.01	1.69±0.10	24.22±1.67	29.73±4.17	28.16±1.19	28.37±1.07	311±9.77
5	75	35	0	0.26±0.01	3.57±0.10	21.78±1.34	25.44±0.84	24.66±1.58	24.86±1.89	448±28.65
6	75	35	30	0.34±0.02	4.32±0.33	29.96±0.45	26.38±0.21	24.13±2.05	24.34±1.91	621±40.11
7	105	35	0	0.09±0.01	1.94±0.15	17.96±0.31	25.33±0.60	28.33±1.07	28.49±1.11	312±24.78
8	105	35	30	0.16±0.01	3.16±0.39	24.84±0.82	21.69±1.03	26.13±1.52	26.34±1.20	359±34.87
9	90	25	0	0.19±0.01	3.11±0.13	23.00±0.92	25.07±1.04	27.25±0.87	27.62±0.99	631±31.33
10	90	25	30	0.23±0.01	4.10±0.18	28.00±0.33	23.27±0.07	25.88±0.66	26.09±0.78	789±36.77

11	90	45	0	0.18±0.01	2.58±0.27	18.57±0.21	27.37±0.34	25.92±0.80	26.2±0.87	289±28.32
12	90	45	30	0.19±0.01	2.44±0.26	26.73±0.48	24.81±0.86	26.00±2.41	26.13±2.10	531±35.45
13	90	35	15	0.15±0.01	2.46±0.08	23.60±1.51	25.81±1.04	25.52±0.85	25.7±0.70	428±23.28
14	90	35	15	0.14±0.01	2.56±0.16	22.84±1.01	23.38±0.82	27.38±1.80	27.63±1.99	431±16.17
15	90	35	15	0.16±0.01	2.98±0.29	22.436±0.21	25.64±0.76	28.06±0.66	28.29±0.78	457±21.11

X_1 = VF temperature (°C), X_2 = VF duration (min), X_3 = Maltose concentration (%);The VF measurement of each samples was performed in triplicate.

Table S3. The predicted results of the central composite rotatable design for VF silver herring.

**Run**	**VF temperature (℃)**	**VF duration (min)**	**maltose concentration (%)**	**Aw**	**moisture content (%)**	**yield (%)**	**oil content(%)**	***L** value**	***ΔE***	**fracturability (g/s)**
1	75	25	15	0.629	7.698	25.240	27.7805	24.648	24.82	279
2	105	25	15	0.206	2.803	20.060	23.6644	30.562	30.73	342
3	75	45	15	0.336	4.740	26.840	29.6233	25.892	26.14	289
4	105	45	15	0.141	1.687	24.220	29.7282	28.162	28.37	311
5	75	35	0	0.259	3.573	21.780	25.4381	24.656	24.86	448
6	75	35	30	0.336	4.317	29.960	26.3776	24.128	24.34	621
7	105	35	0	0.092	1.940	17.960	25.3330	28.326	28.49	312
8	105	35	30	0.157	3.163	24.840	21.6902	26.126	26.34	359
9	90	25	0	0.190	3.113	23.000	25.0691	27.25	27.62	631
10	90	25	30	0.228	4.097	28.000	23.2746	25.88	26.09	789
11	90	45	0	0.179	2.577	18.570	27.3727	25.924	26.20	289
12	90	45	30	0.190	2.437	26.730	24.8138	26.0013	26.13	531
13	90	35	15	0.154	2.460	23.600	25.8091	25.522	25.70	428
14	90	35	15	0.142	2.557	22.840	23.3836	27.378	27.63	431
15	90	35	15	0.164	2.980	22.436	25.6433	28.058	28.29	457

X_1 = VF temperature (°C), X_2 = VF duration (min), X_3 = Maltose concentration (%).
